# Patient preferences for drug therapy in inflammatory arthritis: protocol for a living systematic review and evidence map to inform clinical practice guidelines

**DOI:** 10.1136/bmjopen-2024-088267

**Published:** 2025-01-15

**Authors:** Pakeezah Saadat, Nick Bansback, Marie Falahee, Mickaël Hiligsmann, Peter Tugwell, Rachelle Buchbinder, Samuel Whittle, Dawn P Richards, Laurie Proulx, Holger Schunemann, Pablo Alonso-Coello, Robby Nieuwlaat, Wojtek Wiercioch, Simon Kuper, Jordi Pardo Pardo, Glen Hazlewood

**Affiliations:** 1 Institute of Health Policy, Management, and Evaluation, University of Toronto, Toronto, Ontario, Canada; 2 School of Population and Public Health, The University of British Columbia, Vancouver, British Columbia, Canada; 3 Arthritis Research Canada, Richmond, British Columbia, Canada; 4 Rheumatology Research Group, Institute of Inflammation and Ageing, College of Medical and Dental Sciences, University of Birmingham, Birmingham, UK; 5 NIHR Birmingham Biomedical Research Centre, University Hospitals Birmingham NHS Foundation Trust, Birmingham, Birmingham, UK; 6 Department of Health Services Research, CAPHRI Care and Public Health research Institute, Maastricht University, Maastricht, Netherlands; 7 Department of Medicine, University of Ottawa Faculty of Medicine, Ottawa, Ontario, Canada; 8 Ottawa Hospital Research Institute Clinical Epidemiology Program, Ottawa, Ontario, Canada; 9 Musculoskeletal Health and Wiser Health Care Units, School of Public Health and Preventive Medicine, Monash University, Melbourne, Victoria, Australia; 10 School of Public Health and Preventive Medicine, Monash University, Melbourne, Victoria, Australia; 11 Rheumatology Unit, Queen Elizabeth Hospital, Adelaide, South Australia, Australia; 12 Canadian Arthritis Patient Alliance, Toronto, Ontario, Canada; 13 Department of Health Research Methods, Evidence and Impact, McMaster University, Hamilton, Ontario, Canada; 14 Department of Medicine, McMaster University, Hamilton, Ontario, Canada; 15 Department of Biomedical Sciences, Humanitas University, Milano, Italy; 16 Centro GRADE Barcelona, Instituto de Investigacion Biomedica, Barcelona, Spain; 17 Michael G. DeGroote Cochrane Canada & McMaster GRADE Centres, McMaster University, Hamilton, Ontario, Canada; 18 World Health Organization Collaborating Center for Infectious Diseases, Research Methods and Recommendations, McMaster University, Hamilton, Ontario, Canada; 19 Faculty of Health, Medicine and Life Sciences, Maastricht University, Maastricht, Limburg, Netherlands; 20 Cochrane Musculoskeletal, Faculty of Medicine, University of Ottawa, Ottawa, Ontario, Canada; 21 Departments of Medicine and Community Health Sciences, University of Calgary Cumming School of Medicine, Calgary, Alberta, Canada; 22 McCaig Institute for Bone and Joint Health, University of Calgary Cumming School of Medicine, Calgary, Alberta, Canada

**Keywords:** Patients, RHEUMATOLOGY, Systematic Review

## Abstract

**Abstract:**

**Introduction:**

The pharmacological management of inflammatory arthritis often requires choices that involve trade-offs between benefits, risks and other attributes such as administration route, frequency and cost. This living systematic review aims to inform international clinical guidelines on inflammatory arthritis by creating an evidence map of patient preference studies concerning the trade-offs in pharmacological management of inflammatory arthritis.

**Methods and analysis:**

We will include published and peer-reviewed full-text studies in any language that quantitatively assess preferences of patients for the pharmacological management of inflammatory arthritis (rheumatoid arthritis, spondyloarthritis and juvenile idiopathic arthritis). Studies must use either stated or revealed preference methods to assess preferences and provide a quantitative assessment of relevant characteristics, such as benefits, risks, costs and process attributes. Articles will identified through Medline and EMBASE database searches from inception using search terms that combine keywords and subject headings for inflammatory arthritis and preference-based methods, and a search in the Health Preference Study and Technology Registry using keywords for the populations of interest. Two independent reviewers will perform abstract and full-text screening. Risk of bias will be assessed using the GRADE risk of bias tool. An evidence map will be generated to summarise included studies and their assessments of each trade-off. The search will be conducted every 6 months with new studies added to the inventory.

**Ethics and dissemination:**

Ethics approval is not required. Results from the base review will be published in a peer-reviewed journal and findings will be presented at conferences. In the living model, we will publish updates and datasets on an Open Science Framework page, with periodic updates in peer-reviewed journals.

**STRENGTHS AND LIMITATIONS OF THIS STUDY**

- This living systematic review of patient preference studies will inform international clinical guidelines for inflammatory arthritis.
- Biannual updates will be conducted to promptly integrate emerging evidence, facilitating the maintenance of up-to-date clinical guidelines for inflammatory arthritis.
- Since the field is still evolving, the search strategy and data presentation will be adapted to incorporate newly emerging evidence.
- We will not meta-analyse the relative importance of each attribute as quantitative synthesis poses a challenge due to significant heterogeneity in study designs and attributes.

## Introduction

 Treatment choices in inflammatory arthritis nearly always involve trade-offs. People living with inflammatory arthritis need to choose between treatments that can differ in their benefits, risks and other characteristics such as route of administration, frequency and cost. For example, in rheumatoid arthritis, ’triple therapy’ with methotrexate, sulfasalazine and hydroxychloroquine has additional burden in terms of more pills, potentially more adverse effects, but an improved chance of a treatment response over methotrexate alone.^1 2^ Tofacitinib, a Janus Kinase (JAK) inhibitor, has been shown to have a higher risk of cardiovascular adverse events and certain malignancies in people at risk for cardiovascular events,^3^ but is available as a pill and is an effective option for many patients. For people with psoriatic arthritis, certain treatments may work better for different disease manifestations, such as skin disease or enthesitis, and this needs to be balanced against the control of their joint disease.^4 5^ Corticosteroids offer rapid improvement for many patients with inflammatory arthritis, but long-term use is associated with multiple risks.^6^ Understanding patient preferences for these trade-offs is important to guide patient-centred care.

Patient preferences can be assessed or measured in multiple ways. Qualitative approaches can help understand how patients approach the decision-making process and the relevant considerations for a given healthcare decision.^7^ Quantitative approaches provide numerical estimates of patient preferences for different treatments or treatment attributes, including risks and benefits and other considerations, such as route of administration or cost.^8 9^ Quantitative methods can be categorised into revealed preference methods that measure which treatment people choose when presented with an actual choice, or stated preference methods that ask patients to rate, rank or choose between hypothetical treatment options or attributes. Stated preference methods can be further categorised according to different frameworks.^8 10^ Revealed preference methods, where actual choices of participants are recorded are challenging, as real-world choices are often influenced by the healthcare providers’ recommendations or restrictions imposed by insurance plans. Hence, most studies use stated preference methods.

When developing clinical practice guidelines, it is important to understand patient preferences for the relevant trade-offs. Under the GRADE approach, patient values and preferences are a key consideration in the Evidence to Decision framework, when deciding on the direction and strength of a recommendation.^11^ While not often done, a systematic review of patient preference studies is recommended to inform these judgements.^12^ Quantitative estimates of patient preferences are desired, as guideline panels need to consider which outcomes or other attributes are most important and by how much. When a guideline panel is confident that based on the balance of benefits and harms nearly all patients would choose a particular course of action then a strong recommendation can be made. Otherwise, a conditional recommendation is made, either for or against the treatment.

We have recently started living clinical guidelines for inflammatory arthritis in both Canada^13 14^ and Australia.^15^,^17^ In a living clinical practice guideline, treatment recommendations are kept up to date as new evidence emerges.^18^ Within an entire guideline, individual recommendations may remain stable, while others may be updated in a living mode through living systematic reviews of the risks and benefits, which we have initiated for inflammatory arthritis.^19^ Each time a recommendation is added, or new evidence emerges, guideline panels need to either make or update their judgements regarding the balance of risks and benefits. In a living model, this requires regular updates of the evidence on patient preferences. New preference studies may have been published, and treatments may have new attributes, such as newly discovered risks. For example, in our inflammatory arthritis guidelines, our recommendation for choices of treatment after an inadequate response to anti-TNF therapy^14 15^ required an update to our prior systematic review on patient preferences,^20 21^ given the risks of malignancy and cardiovascular events with JAK inhibitors. We did not find any studies that measured preferences for these trade-offs, but this evidence will likely emerge over time.

The aim of this systematic review will be to develop a living evidence map of patient preference studies as they relate to the treatment of inflammatory arthritis. By ‘evidence map’ we mean a catalogue of studies that are characterised in terms of their characteristics and methods used, risk of bias, and which outcomes or other treatment attributes they included. We do not intend to summarise or meta-analyse the relative importance of each attribute, which is often challenging due to study heterogeneity. Rather, the intention is that guideline developers can use the evidence map to identify and review the studies relevant to their context to help inform clinical guideline recommendations.

## Methods and analysis

This protocol adheres to the Preferred Reporting Items for Systematic Reviews and Meta-Analyses Protocols (PRISMA-P) checklist,^22^ which is available in the supplementary material.

### Eligibility criteria

#### Population

We will include any study that provides a quantitative assessment of patients’ preferences for the management of inflammatory arthritis. Inflammatory arthritis includes rheumatoid arthritis, juvenile idiopathic arthritis and spondyloarthritis, as defined by the study authors. Spondyloarthritis includes psoriatic arthritis, ankylosing spondylitis, reactive arthritis, enteropathic arthritis, and axial and/or peripheral spondyloarthritis not otherwise classified. We will include studies that have at least 75% of the included participants with inflammatory arthritis.

#### Outcomes

To be included, a study must include a quantitative assessment of the importance of attributes relevant to the pharmacological and non-pharmacological management of inflammatory arthritis. This includes treatment benefits, risks, and process attributes. Process attributes include any aspect related to care delivery, such as route and frequency of administration, access to care and costs. Preferences may be assessed for attributes separately, or together, as would be the case when presenting patients with a ‘real-world’ choice between treatment options that differ across multiple characteristics (ie, in revealed preference studies). We will exclude studies that exclusively provide an estimate of patients’ health-related quality of life (HRQOL). HRQOL measures the value a patient places on their current health state and not their preference for potential treatment outcomes or attributes.^9 23^

#### Study design

We will include any published and peer-reviewed full-text study in any language that assessed preferences using stated-preference methods^10^ (where participants are asked their preferences for hypothetical choices) or revealed-preference methods (where the actual choices of patients are observed after being presented with a decision-aid).^8^ We will exclude abstracts, pre-print articles and studies that have not been peer-reviewed. The stated-preference methods categorised by Soekhai *et al*^10^ consist of four distinct categories (table 1). Discrete-choice experiments examine trade-offs between attributes and their alternatives given a series of choice sets. Ranking methods are used to elicit an order of attributes and their alternatives through ranking exercises, such as best-worst scaling. Indifference methods ask people to make a choice between staying in a given health state for the rest of their life versus a return to full health with a shortened life expectancy (time trade-off) or a gamble with a chance of returning to full health but also a chance of immediate death (standard gamble). The thresholds (life expectancy or chance of death) are manipulated until the point of indifference is found. Rating methods ask people to choose the strength of their preference using a labelled scale.

**Table 1:** Inventory of patient-preference methods (adapted from Soekhai *et al*^10^)

Category	Method
Discrete-choice-based methods	Discrete choice experiment/best-worst scaling (type 3)
Adaptive conjoint analysis
Ranking methods	Qualitative discriminant process
Q-methodology
Control preferences scale
Best-worst scaling (types 1 and 2)
Self-explicated conjoint
Indifference methods	Standard gamble
Time trade-off
Person trade-off
Starting known efficacy
Test trade-off
(Probabilistic) threshold technique
Contingent valuation
Rating methods	Constant sum scaling
Repertory grid method
Analytic hierarchy process
Swing weighting
Visual analogue scale
Allocation of points
Outcome prioritisation tool
Measure of value
Revealed preferences	Patient preference trials
Direct questions in clinical trials

*Living review considerations*: If new preference-based approaches are identified or developed over time, these will be added to the eligibility criteria. Our search (described below) is also designed to identify qualitative studies, which would allow us to expand the scope of work in the future.

### Information sources and search strategy

We will search the following databases from inception: Medline In-Process and Other Non-Indexed Citations, and EMBASE (Excerta Medica Database). The search strategy is presented in online supplemental appendix A and combines keywords and subject headings for inflammatory arthritis and preference-based methods. The MEDLINE and EMBASE inflammatory arthritis filters were derived from Cochrane reviews and adapted for the other databases. The preference-based method filter was derived from Selva *et al*^24^ and supplemented with additional filters to capture all methods in table 1, as well as qualitative studies. Additionally, we will search the Health Preference Study and Technology Registry (HPSTR; https://hpstr.org/), a web-based registry of health preference studies and technologies, using keywords for each population of interest (defined above).

*Living review considerations*: The search filters will be reviewed periodically and updated as needed, particularly if new validated filters for preference studies are published that suit our purpose. For the base review, the search will be conducted once, then updated prior to publication, after which the review will transition to a living mode. In the living mode, the search will be updated every 6 months, with the frequency adjusted as needed based on the usefulness and feasibility.

### Article screening

**Figure 1:**
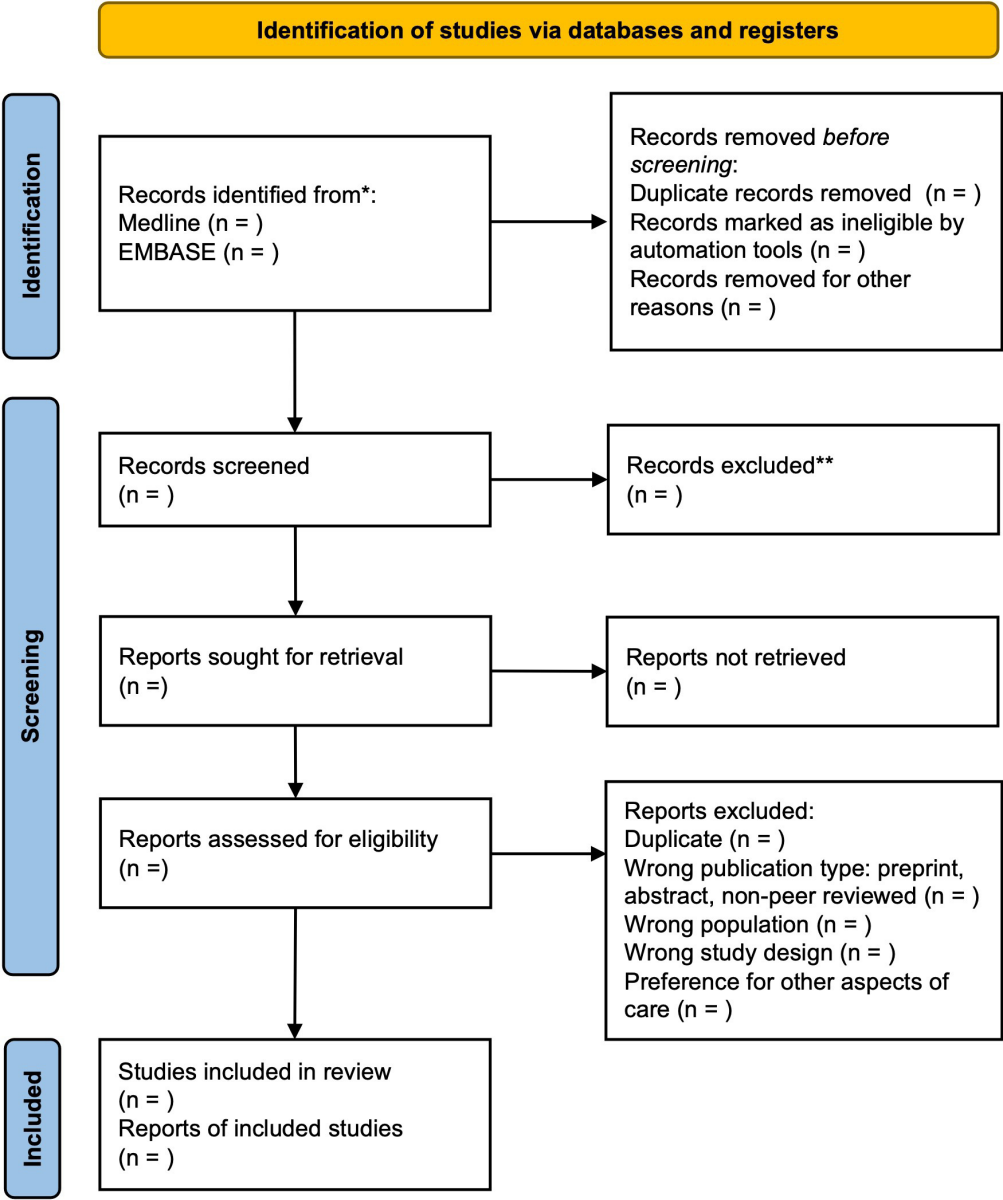
PRISMA flow diagram for the living systematic review of patient preference studies in inflammatory arthritis.

The titles and abstracts of all records will first be screened for eligibility independently by two reviewers. Any record that either reviewer marks as unclear or included will proceed to full-text review. Full-text review will also be done by pairs of reviewers working independently. Any disagreement at the full-text stage will be discussed between reviewers and with senior reviewer(s) as necessary. At the full-text stage, articles will be excluded in the following hierarchy: wrong publication type (eg, pre-print, abstract); wrong population; wrong study design (not a preference-elicitation method); preferences for other aspects of care (eg, diagnostic approaches). Articles in a different language will be translated into English. An example PRISMA flow-chart is presented in figure 1.

*Living review considerations*: We will explore the incorporation of technologies to improve the efficiency of the article screening process, including automation, machine learning and crowd sourcing. We have employed these tools in other reviews of interventions,^19 25^ but to our knowledge they have not yet been developed for patient preference literature. It is our hope that the results of our review could be used to develop automated approaches for the screening of records.

### Data extraction

Study data will be extracted independently by two team members. From each study we will extract relevant study and participant characteristics as listed in online supplemental appendix B if reported. Study characteristics include details on the population of interest, setting, response rate, recruitment strategy, funding, statistical analysis used, attribute selection process and preference-elicitation methods. Participant characteristics include age, sex/gender, disease duration and severity, health literacy, and sociodemographic characteristics, particularly those that would identify patient populations at risk for inequities in care. In prior studies, we have identified the following seven priority populations for guidelines in rheumatoid arthritis:^26 27^ rural and remote residents, indigenous peoples, elderly persons with frailty, first-generation immigrants and refugees, persons of low socioeconomic status or who are vulnerably housed, sex and gender diverse populations, and Black Canadians (added since our original framework.^28^

For each study, we will extract the attributes and levels evaluated, including a short description and the actual description presented in the survey to participants. The results of the study will be extracted as the estimated value for each attribute value, including the point estimate (mean/median), scale and measure of variability. If this data is not presented it will calculate from the available data, if possible. Otherwise, we will extract the data as reported by the study (eg, relative importance, ranks of attribute importance). We will extract measures of variability in the following order depending on availability: SDs, SEs, 95% CIs, or CrI, and exact *p* values with the statistical test used. Data screening and extraction will be done via Covidence systematic review software (Veritas Health Innovation, Melbourne, Australia, available at www.covidence.org).

*Living review considerations*: Depending on the volume of studies identified, the timing of data extraction for each study may need to be staged, prioritising those studies that are most relevant to inform our living guidelines. We will include a table of studies pending data extraction and risk of bias assessment in the review.

### Risk of bias

The risk of bias in each study will be assessed using the GRADE risk of bias tool for value and preference studies, which has been developed and validated, and is being finalised for publication. Assessment will be done independently by two team members and any discrepancies will be resolved through consensus.

*Living review considerations*: If preferred risk of bias tools change over time, these will be incorporated in the review.

### Data presentation

Study characteristics will be summarised descriptively in a table. An evidence map will be generated that summarises the current literature in terms of which available studies assess each trade-off. An example is included in table 2. The rows and columns will contain all attributes assessed. Attributes will be grouped into categories by two independent reviewers with help from a senior reviewer, if needed. Each cell will list the study(s) that included those attributes. Studies that include absolute anchors of attribute importance (eg, measured on a 0–1 scale, where 0 represents death and 1 represents full health), such as a standard gamble, time trade-off or visual analogue scales, will be included in the bottom row. This would allow guideline panels to easily identify potential studies that would be relevant for their purpose and help identify gaps in the evidence.

**Table 2:** Example of the evidence map displaying studies that have included each pair of attributes

		Benefits		Risks		Process	
		Remission	Pain reduction	GI side effects	Serious side effects	Route of delivery	Cost
Benefits	Remission	–	–	–	–	–	–
	Pain reduction	(Study IDs)	–	–	–	–	–
Risks	Gastrointestinal side effects	(Study IDs)	(Study IDs)	–	–	–	–
	Serious side effects	(Study IDs)	(Study IDs)	(Study IDs)	–	–	–
Process	Route of delivery	(Study IDs)	(Study IDs)	(Study IDs)	(Study IDs)	(Study IDs)	–
	Cost	(Study IDs)	(Study IDs)	(Study IDs)	(Study IDs)	(Study IDs)	–
	Absolute anchor of importance	(Study IDs)	(Study IDs)	(Study IDs)	(Study IDs)	(Study IDs)	(Study IDs)

*Living review considerations*: We will explore the value of including additional information within each cell of the evidence map (eg, sample size, risk of bias), and alternative ways of summarising the data to guideline panels, including interactive evidence maps that would provide more flexibility.

### Patient and public involvement

This protocol was reviewed and refined with feedback from our patient partners, DPR and LP, who are individuals living with rheumatoid arthritis/juvenile idiopathic arthritis.

## Ethics and dissemination

Ethics approval is not required. Results from the base review will be published in a peer-reviewed journal and findings will be presented at conferences. In the living model, we will publish updates and datasets on an Open Science Framework page, with periodic updates in peer-reviewed journals.

## supplementary material

10.1136/bmjopen-2024-088267 online supplemental file 1
