# Supplementary material for: Patient preferences for drug therapy in inflammatory arthritis: protocol for a living systematic review and evidence map to inform clinical practice guidelines
Source: BMJ Open. 2025 Jan 15;15(1):e088267. doi: 10.1136/bmjopen-2024-088267 (PMC11751828; doi:10.1136/bmjopen-2024-088267)
Supplement: online supplemental file 1 [file bmjopen-15-1-s001.docx]

Table of Contents

[**Appendix A. Search strategy (adapted from Selva et al, 2017)** 2](#_Toc137818663)

[**Appendix B. List of variables to be extracted** 5](#_Toc137818664)

# **Appendix A. MEDLINE Search strategy (adapted from Selva et al, 2017)^[[1]](#footnote-1)^**

**# Query**

1 exp arthritis, rheumatoid/

2 ((rheumatoid or reumatoid or revmatoid or rheumatic or reumatic or revmatic or rheumat* or reumat* or revmarthrit*) adj3 (arthrit* or artrit* or diseas* or condition* or nodule*)).tw.

3 1 or 2

4 exp Arthritis, Juvenile Rheumatoid/

5 JIA.tw

6 (juvenile adj2 arthritis).tw.

7 or/4-6

8 enthesitis.tw.

9 Arthritis, Psoriatic/

10 oligoarthritis.tw.

11 or/8-10

12 (child* or adolescent* or infan*).tw.

13 11 and 12

14 limit 11 to (infant or child or preschool child <1 to 6 years> or school child <7 to 12 years> or adolescent <13 to 17 years>)

15 13 or 14

16 Spondylarthritis/

17 spondylarthritis.ti,ab.

18 spondyloarthritis.ti,ab.

19 Spondylarthropathies/

20 spondylarthropath$.ti,ab.

21 spondyloarthropath$.ti,ab.

22 Spondylitis/

23 Spondylitis, Ankylosing/

24 ankylosing spondylitis.ti,ab.

25 spondylitis.ti,ab.

26 ((axial adj (SpA or disease or arthritis)) or axial joint disease).ti,ab.

27 enthesitis.ti,ab.

28 sacroiliitis/ or (sacroiliitis or sacroilitis).ti,ab.

29 (peripheral adj2 arthritis).ti,ab.

30 Or/16-29

31 Arthritis, Psoriatic/

32 (psoria$ adj (arthriti$ or arthropath$)).tw.

33 ((arthriti$ or arthropath$) adj psoria$).tw.

34 Or/29-31

35 3 or 15 or 34

36 qualitative stud*.tw.

37 exp Qualitative Research/

38 survey*.tw.

39 exp Data Collection/

40 questionnaire*.tw.

41 focus group*.tw.

42 conjoint analysis.tw.

43 discrete choice experiment*.tw.

44 rating task*.tw.

45 ranking task*.tw.

46 choice experiment*.tw.

47 decision aid*.tw.

48 risk attitude*.tw.

49 risk aversion.tw.

50 discrete choice*.tw.

51 standard gamble.tw.

52 willingness to pay.tw.

53 willingness-to-pay.tw.

54 decision support technique*.tw.

55 decision support system*.tw.

56 decision making.tw.

57 time trade*.tw.

58 exp Questionnaires/

59 trade off*.tw.

60 stated preference*.tw.

61 contingent valuation.tw.

62 choice experiment.tw.

63 best-worst scaling.tw.

64 Q-method*.tw

65 control preference* scale.tw.

66 self-explicated conjoint.tw.

67 Start* known efficac*.tw.

68 threshold technique.tw.

69 constant sum scal*.tw.

70 repertory grid method.tw.

71 exp Analytic Hierarchy Process/

72 swing weight*.tw.

73 visual analog* scale.tw.

74 allocat* of point*.tw.

75 outcome prioriti* tool.tw.

76 measure of value.tw.

77 preference trial.tw.

78 grounded theory/ or qualitative research/

79 (qualitative* or focus group* or interview* or mixed method* or mixed-method* or content analysis or thematic analysis or phenomenological study or ethnograph* or interpretive description or narrative* or semi-structured or unstructured or face-to-face or constant comparative or participant observation or audio recorded).mp.

80 px.fs

81 or/36-80

82 exp Consumer Satisfaction/

83 exp Consumer Participation/

84 exp Patient Satisfaction/

85 patient perspective*.tw.

86 exp "Attitude of Health Personnel"/

87 exp Health Knowledge, Attitudes, Practice/

88 exp "Delivery of Health Care"/

89 patient compliance.tw.

90 patient participation.tw.

91 patient satisfaction.tw.

92 treatment refusal.tw.

93 patient preference*.tw.

94 patient opinion*.tw.

95 patient belief*.tw.

96 patient concern*.tw.

97 patient perspective*.tw.

98 patient choice*.tw.

99 patient value*.tw.

100 patient priorit*.tw.

101 exp Health Priorities/

102 patient perception*.tw.

103 choice behavio*.tw.

104 patient consensus.tw.

105 exp Consensus/

106 (dissent and dispute*).tw.

107 uncertaint*.tw.

108 (utility or utilities).ti,ab.

109 discrete choice*.tw.

110 ((patient$ or participant$) adj3 (participation or satisfaction or perspective$ or compliance or preference$ or opinion$ or belief$ or concern$ or choice$ or value$ or priorit$ or perception$ or request$)).tw.

111 or/82-110

112 35 and 81 and 111

113 exp animals/ not humans.sh.

114 112 not 113

**Appendix B. List of variables to be extracted**

| **Variable** | **Explanation** | **Response format** | **Notes** | **Examples** |
| --- | --- | --- | --- | --- |
| **Study characteristics** | | | | |
| Method | How were the preferences elicited | Free text | Use terminology from Table 1 where possible. Include all methods if more than one used. | -Discrete-choice experiment  -Standard gamble; simple direct weighting (visual analogue scale) |
| Attribute selection process | How were the attributes selected | Free text |  | -Qualitative study  -Chosen by experts |
| Treatments of interest | Which treatments were the focus of the study | Free text |  | -csDMARDs, bDMARDs, tofacitinib  -corticosteroids |
| Country(s) | Which country(s) was the study conducted in | Free text | If > 5 countries, summarize according to continent or geographic region. List countries alphabetically. | -Finland, Sweden |
| Setting | Clinic setting or sampling framework | Free text | Describe where patients in the study were sampled from | -Outpatient clinics at one academic centre  -Outpatient clinics at academic and community centres  -Patient registry  -Online panel |
| Recruitment procedure | How were people recruited | Free text | Describe the method of recruitment | -In person clinic recruitment  -E-mail to patients in an existing registry  -Link to the study shared through patient groups |
| Eligibility criteria | Eligibility criteria for the study | Free text | Copy verbatim from the study description |  |
| Patient Population | Health conditions of the population studied | Free text | Include the percentages if a mixed population | RA (45%), PsA (30%), Ankylosing spondylitis (25%) |
| Sample size | Number of people who completed the survey or study | Numeric | If some participants had missing data and this is reported, record the number of people who completed the preference-elicitation aspect of the study |  |
| Response rate (offered) | Percentage of people who were offered the study that completed it | Numeric | In a study where people were recruited in clinic or through a registry, this would be the number approached (for example, either in person or through e-mail) who completed the study.  In a study where a link to the study was posted on a patient website, it would not be possible to estimate this number. |  |
| Response rate (consented/viewed) | Percentage of people who consented or viewed the study that completed it | Numeric | Percentage of people who consented to the study, or who started the study (for example, in the case of an e-mailed link) and completed it. |  |
| Funding | Funding received for the study | Free text | List the funding agency or company name | -Public (funding agency)  -Industry (company name)  -Public and industry (company name) |
| Industry affiliations of authors | Are authors employees or affiliated with a pharmaceutical company | Free text | Yes/no, then list affiliation(s) | -No  -Yes (company name) |
| Statistical analysis | Statistical method used to analyse data | Free text | List the method as stated in the text | -Mixed logit model  -Hierarchical bayesian |
| **Patient characteristics** | | | | |
| Age | Mean or median age of study participants | Numeric | If both mean and median reported, record mean unless sample skewed |  |
| Sex/Gender | The sex and/or gender of the participants. | Free text with percentage values | Record as recorded/ reported by the study. Include all gender categories that were response options, if available. If both sex and gender reported, describe both. | -Female (89%), male (11%), non-binary (0.2%), prefer not to answer (0.5%) |
| Disease duration (years) | How many years have the people in the study had their disease | Numeric | Record as reported by the study. In mixed populations, record for each disease, if available | -10.7  -5.5 (RA); 6.3 (PsA) |
| Disease severity | Measures of disease severity, relevant to the population studied | Free text | Any validated measure of disease severity e.g., DAS28, CDAI, BASDAI. Extract all that are available. |  |
| Physical function | Measures of functional status | Free text | Any validated measure of disease severity e.g., HAQ-DI, PROMIS Physical Function |  |
| Ethnicity | Ethnicity of study participants | Free text with percentage values | Record as reported by the study | -Caucasian (55%); Black (20%); Hispanic (10%); other (15%) |
| Education | Education level of study participants | Free text with percentage values | Record as reported by the study | -Greater than high-school (55%) |
| Place of residence of participants | Any details on the location of participants (e.g., urban/rural) | Free text with percentage values | Only record if directly reported by the study i.e., do not assume that if the study is conducted at an academic centre, the participants are urban. | -Urban (75%); rural (25%)  -Immigrant/ refugee/ vulnerably housed. |
| Health literacy | Any measure of the health literacy of participants | Free text | Record as reported by the study. Some tools for measuring are [here](https://www.cdc.gov/healthliteracy/researchevaluate/measure-peoples-skills-experiences.html). |  |
| Additional patient characteristics relevant for the study | Any additional characteristics relevant to the study | Free text | For example, a study may have a particular focus on a subgroup of patients and could report those characteristics here if not otherwise captured above. |  |

1. Selva A, Sola I, Zhang Y, et al. Development and use of a content search strategy for retrieving studies on patients' views and preferences. Health Qual Life Outcomes 2017;15:126. [↑](#footnote-ref-1)
