# Supplementary material for: Patient preferences for drug therapy in inflammatory arthritis: protocol for a living systematic review and evidence map to inform clinical practice guidelines
Source: BMJ Open. 2025 Jan 15;15(1):e088267. doi: 10.1136/bmjopen-2024-088267 (PMC11751828; doi:10.1136/bmjopen-2024-088267)
Supplement: Supplementary file 2 [file bmjopen-2024-088267.reviewer_comments.pdf]

PEER REVIEW HISTORY

BMJ Open publishes all reviews undertaken for accepted manuscripts. Reviewers are asked to complete a checklist review form and are provided with free text boxes to elaborate on their assessment. These free text comments are reproduced below.

ARTICLE DETAILS

Title (Provisional)

Patient preferences for drug therapy in inflammatory arthritis: protocol for a living systematic review and evidence map to inform clinical practice guidelines

Authors

Saadat, Pakeezah; Bansback, Nick; Falahee, Marie; Hiligsmann , Mickaël; Tugwell, Peter; Buchbinder, Rachelle; Whittle, Samuel; Richards, Dawn P.; Proulx, Laurie; Schunemann, Holger; Alonso-Coello, Pablo; Nieuwlaat, Robby; Wiercioch, Wojtek; Kuper, Simon; Pardo Pardo, Jordi; Hazlewood, Glen

---

VERSION 1 - REVIEW

**Reviewer**	**1**
**Name**	**Conti, Valeria**
**Affiliation**	**University of Salerno**
**Date**	**30-Jul-2024**
**COI**	**None**

A growing number of studies demonstrate the importance of considering patients' opinions about the drug treatments they undergo during their course of treatment. Indeed, it is also important to assess their approach when faced with therapeutic choices they have already made and their preferences among different treatments (taking into account the different characteristics of these treatments, such as the route of administration, associated benefits and risks). This will allow, in clinical practice, to arrive at more effective and targeted therapeutic decisions that will ensure a better quality of life. One of the primary objectives of the following work is to produce an “evidence map” through a systematic review of the existing literature on patient preferences in the pharmacological management of inflammatory arthritis, thus updating existing clinical practice guidelines on the same condition.

I have the following concerns:

The protocol is well written, with a fairly thorough consultation of the most recent literature. However, more editing is needed in the English language. In addition, I suggest reviewing the formatting of the entire manuscript, as two mismatched page numbering methods were used (e.g., on page 1 of 31, the wording in the upper right corner does not match the page number in the lower right corner, and on page 22 of 31, the numbering in the lower right corner starts again from number 1).

Abstract:

The pharmacological management of inflammatory arthritis often requires individuals to make choices that involve trade-offs

Please change to ...”requires choices that involve..”

INTRODUCTION

Page 5 of 31, lines 20-25

-Write the abbreviation JAK in full when it is first mentioned.

Page 5 of 31, lines 4-35 and 37-60

“Corticosteroids offer rapid improvement for many patients with inflammatory arthritis, but long-term use is associated with multiple risks.”

“Patient preferences can be assessed or measured in multiple ways. Qualitative approaches can help understand how patients approach the decision-making process and the relevant considerations for a given healthcare decision.”

-These two paragraphs of the introduction are not well connected. Considering what has already been said in lines 4-35, I suggest adding before lines 37-60:

“Therefore, as rheumatoid arthritis can be managed with different pharmacological classes and with different risk/benefit profiles, it would be good to take into account patients’ preferences and personal assessments, once informed of all the characteristics of possible pharmacological treatments (in terms of therapeutic response, tolerability, route of administration, etc.)”

Page 6 of 31, lines 45-50

“Within an entire guideline, individual recommendations may remain stable, whereas others may be updated in a living mode through living systematic reviews of the risks and benefits, which we have initiated for inflammatory arthritis.”

-I suggest rewording the sentence above with: “Within an entire guideline, individual recommendations may remain stable, while others may be permanently updated through systematic reviews of the risks and benefits, which we have initiated for inflammatory arthritis.”

METHODS

Page 8 of 31, line 45

“This protocol adheres to the PRISMA-P checklist.”

-No reference is made to the PRISMA-P Checklist table in the text, which you inserted at the end of your protocol. I suggest inserting a reference to the figure in the text, perhaps as an additional appendix in the “Table of Contents” section.

Study Design

Page 10 of 31, lines 8-13

“Indifference methods, such as time trade-off or standard gamble, manipulate attribute values until the participant is indifferent, or has no preference.”

-The above-mentioned period belongs to the Study Design subsection of the Methods, in which the four categories of the “Stated Preference Methods” are presented. Reading about the “Indifference Methods”, one of the four categories, it is not immediately clear what is required of the patient (it was necessary to consult the scientific article that you have used as a reference). I suggest further clarification of this part.

Information sources and search strategy

Page 12 of 31, lines 18-25

“Additionally, we will search the Health Preference Study and Technology Registry (HPSTR; https://hpstr.org/), a web-based registry of health preference studies and technologies, using keywords for each population of interest (defined above).”

-The guidelines for authors, from the journal BMJ, specify that protocols must include certain elements, including the date of the study. In the section “Sources of information and search strategy”, after describing how the search will be carried out on the various databases, I strongly suggest adding: “The search will be carried out after the publication of this protocol (estimated to start on (DD/AA/MM) and will be completed on (DD/AA/MM).”

Data presentation

Page 15 of 31, lines 23-25

“Studies that include absolute anchors of attribute importance, such as a standard gamble, time trade-off or visual analogue scales, will be included in the bottom row.”

-Also in Table 2, an example of an evidence map of the studies that will be included, the phrase “absolute anchor of importance” has been inserted in one of the cells. What is meant by “absolute anchor of importance”? I suggest clarifying this part of the methods, because it is not immediately comprehensible.

Funding Statement

Page 17 of 31, lines 53-60

-The guidelines for authors, from the journal BMJ, specify that protocols must include certain elements, including the section “Competing Interests Statement”. I strongly recommend to add it.

REFERENCES

Page 18 of 31

1. Hazlewood GS, Barnabe C, Tomlinson G, et al. Methotrexate monotherapy and methotrexate

combination therapy with traditional and biologic disease modifying antirheumatic

drugs for rheumatoid arthritis: abridged Cochrane systematic review and network metaanalysis.

BMJ 2016;353:i1777. doi: 10.1136/bmj.i1777 [published Online First: 2016/04/23]

-The way references are written needs to be reviewed. Delete the bold part of the reference.

VERSION 1 - AUTHOR RESPONSE

See attached cover letter. Additionally, we have addressed the additional edits per the Editorial office:

1) Please include figure legends at the end of your main document file.

Response: Done

2) Your tables are too large to be included in the manuscript, specifically table 2. Please either reduce their size (maximum 2 pages / 8 columns) or upload them as supplementary material (please ensure you remove your original table and update the table citations within the manuscript).

Response: We edited Table 2 to have 8 columns. The table is illustrative in nature, so deleting these columns did not impact the content of the manuscript.

3) We note that your funders listed on the system and main document do not match. Please check and amend as needed noting that all information must be identical - including award IDS and grant numbers.

Response: We have made this correction to the online system.

4) Please ensure that all authors are included in your author contributor statement. "Nieuwlaat, Robby" may be misspelled. Please ensure the contributor statement in your manuscript is the same as the statement included in the online submission system. For further information please see https://authors.bmj.com/policies/bmj-policy-on-authorship/

Response: We have verified the contributor statement in both the paper and online system and the spelling of all authors.

Reviewer #1

Comment 1
The protocol is well written, with a fairly thorough consultation of the most recent literature. However, more editing is needed in the English language. In addition, I suggest reviewing the formatting of the entire manuscript, as two mismatched page numbering methods were used (e.g., on page 1 of 31, the wording in the upper right corner does not match the page number in the lower right corner, and on page 22 of 31, the numbering in the lower right corner starts again from number 1).

Response: We have deleted the lower right number; the top right number is added by the manuscript submission system.

Comment 2
Abstract:
The pharmacological management of inflammatory arthritis often requires individuals to make choices that involve trade-offs
Please change to ...”requires choices that involve..”
Response: We have made this edit.

Comment 3
INTRODUCTION
Page 5 of 31, lines 20-25
-Write the abbreviation JAK in full when it is first mentioned.
Response: We have made this edit.

Comment 4
Page 5 of 31, lines 4-35 and 37-60
“Corticosteroids offer rapid improvement for many patients with inflammatory arthritis, but long-term use is associated with multiple risks.”
“Patient preferences can be assessed or measured in multiple ways. Qualitative approaches can help understand how patients approach the decision-making process and the relevant considerations for a given healthcare decision.”

-These two paragraphs of the introduction are not well connected. Considering what has already been said in lines 4-35, I suggest adding before lines 37-60:
“Therefore, as rheumatoid arthritis can be managed with different pharmacological classes and with different risk/benefit profiles, it would be good to take into account patients’ preferences and personal assessments, once informed of all the characteristics of possible pharmacological treatments (in terms of therapeutic response, tolerability, route of administration, etc.)”
Response: We have added the following sentence at the end of the first paragraph to better link these paragraphs: “Understanding patient preferences for these trade-offs is important to guide patient-centered care.”

Comment 4
Page 6 of 31, lines 45-50
“Within an entire guideline, individual recommendations may remain stable, whereas others may be updated in a living mode through living systematic reviews of the risks and benefits, which we

have initiated for inflammatory arthritis."
-I suggest rewording the sentence above with: "Within an entire guideline, individual recommendations may remain stable, while others may be permanently updated through systematic reviews of the risks and benefits, which we have initiated for inflammatory arthritis."
Response: We have changed the word "whereas" to "while", but have left the rest of the sentence the same. "Updated in a living mode" is accepted terminology when referring to living guidelines, and has a different meaning than "permanently", as there is typically a transition out of the living mode at some point.

Comment 5
METHODS
Page 8 of 31, line 45
"This protocol adheres to the PRISMA-P checklist."
-No reference is made to the PRISMA-P Checklist table in the text, which you inserted at the end of your

protocol. I suggest inserting a reference to the figure in the text, perhaps as an additional appendix in the "Table of Contents" section.
Response: We edited this to read: "This protocol adheres to the PRISMA-P checklist, which is available in the supplementary material[22]."

Comment 6
Study Design
Page 10 of 31, lines 8-13
"Indifference methods, such as time trade-off or standard gamble, manipulate attribute values until the participant is indifferent, or has no preference."
-The above-mentioned period belongs to the Study Design subsection of the Methods, in which the four categories of the "Stated Preference Methods" are presented. Reading about the "Indifference Methods", one of the four categories, it is not immediately clear what is required of the patient (it was necessary to consult the scientific article that you have used as a reference). I suggest further clarification of this part. **Response**: We have made edits to better describe these methods: "Indifference methods ask people to make a choice between staying in a given health state for the rest of their life versus a return to full health with a shortened life expectancy (time trade-off) or a gamble with a chance of returning to full health but also a chance of immediate death (standard gamble). The thresholds (life expectancy or chance of death) are manipulated until the point of indifference is found."

Comment 7
Information sources and search
strategy Page 12 of 31, lines 18-
25
"Additionally, we will search the Health Preference Study and Technology Registry (HPSTR; https://hpstr.org/), a web-based registry of health preference studies and technologies, using keywords for each population of interest (defined above)."
-The guidelines for authors, from the journal BMJ, specify that protocols must include certain elements, including the date of the study. In the section "Sources of information and search strategy", after describing how the search will be carried out on the various databases, I strongly suggest adding: "The search will be carried out after the publication of this protocol (estimated to start on (DD/AA/MM) and will be completed on (DD/AA/MM)."
Response: As this is a living review we do not believe specifying an exact date would add useful information. We specify that "for the base review, the search will be conducted once, then updated prior to publication, after which the review will transition to a living mode. In the living

mode, the search will be updated every 6 months, with the frequency adjusted as needed based on the usefulness and feasibility."

Comment 8
Data presentation
Page 15 of 31, lines 23-25
"Studies that include absolute anchors of attribute importance, such as a standard gamble, time trade-off or visual analogue scales, will be included in the bottom row."
-Also in Table 2, an example of an evidence map of the studies that will be included, the phrase "absolute anchor of importance" has been inserted in one of the cells. What is meant by "absolute anchor of importance"? I suggest clarifying this part of the methods, because it is not immediately comprehensible. **Response**: We have edited this in the text to clarify: "Studies that include absolute anchors of attribute importance *(e.g. measured on a 0-1 scale, where 0 represents death and 1 represents full health)*".

Comment 9
Funding Statement
Page 17 of 31, lines 53-60
-The guidelines for authors, from the journal BMJ, specify that protocols must include certain elements, including the section "Competing Interests Statement". I strongly recommend to add it.
Response: We have added this section at the end.

Comment 10
REFERENCES
Page 18 of 31
1. Hazlewood GS, Barnabe C, Tomlinson G, et al. Methotrexate monotherapy and methotrexate combination therapy with traditional and biologic disease modifying antirheumatic
drugs for rheumatoid arthritis: abridged Cochrane systematic review and network metaanalysis. BMJ 2016;353:i1777. doi: 10.1136/bmj.i1777 [published Online First: 2016/04/23]
-The way references are written needs to be reviewed. Delete the bold part of the reference.
Response: We have made this edit.
